# Supplementary material for: Predicting visual function by interpreting a neuronal wiring diagram
Source: Nature. 2024 Oct 2;634(8032):113–23. doi: 10.1038/s41586-024-07953-5 (PMC11446822; doi:10.1038/s41586-024-07953-5)
Supplement: Supplementary file 6 — CRF and ERF predictions for individual TmY4 and TmY9 cells. Analogous to Supplementary Data 3, but for TmY target types. Shown are the top four monosynaptic pathways, the strongest pathway passing through each of the top ten intermediary types (ranking from Extended Data Fig. 7), and the trisynaptic pathway Tm1–TmY–Dm3–TmY (see the section entitled Prediction of spatial normalization). [file 41586_2024_7953_MOESM6_ESM.zip › DataS4/TmY9qΓèÑ/720575940627222683.pdf]

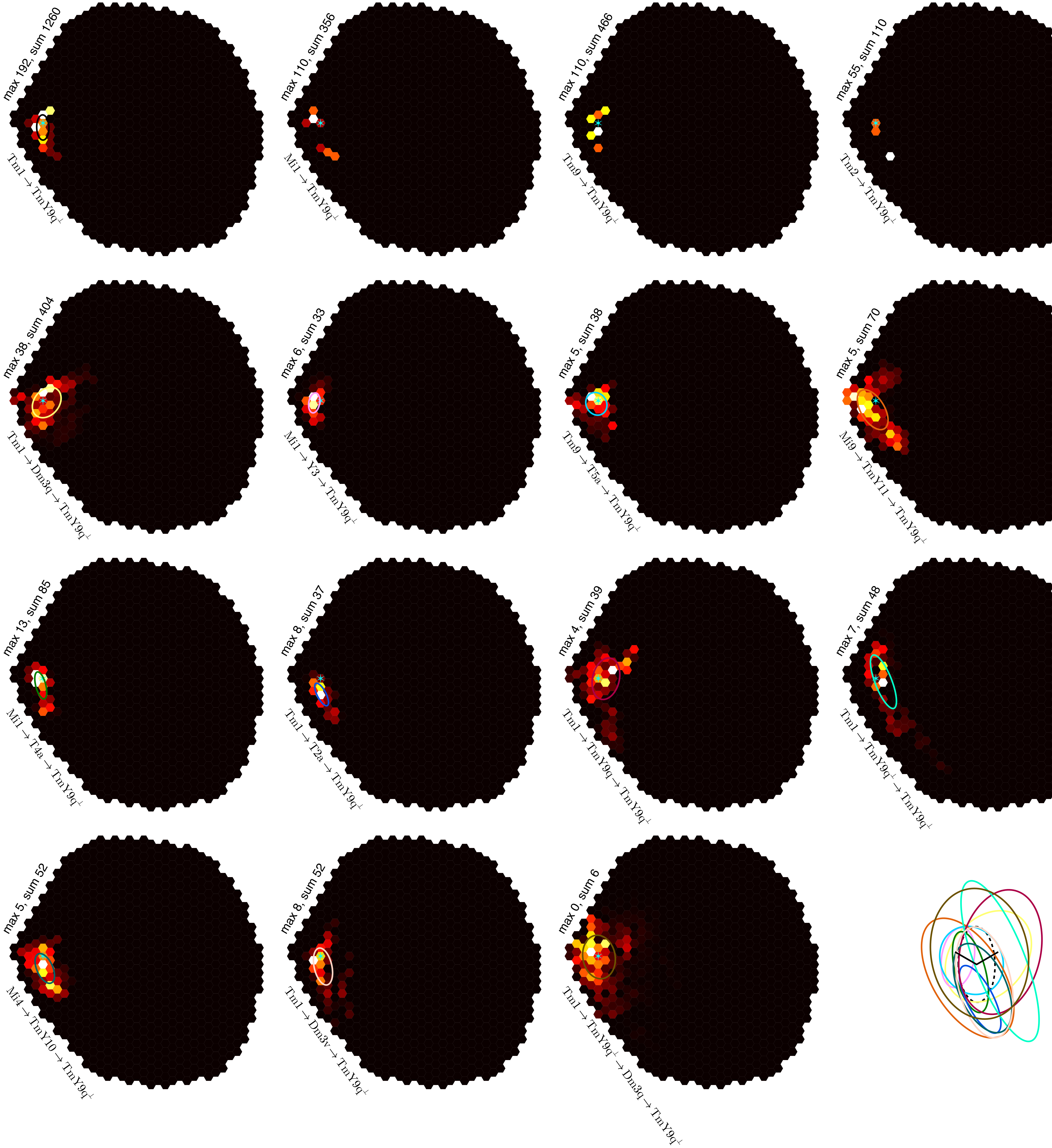
max 192, sum 1260
Tm1 → TmY9q$^{\perp}$
max 110, sum 356
Mi1 → TmY9q$^{\perp}$
max 110, sum 466
Tm9 → TmY9q$^{\perp}$
max 55, sum 110
Tm2 → TmY9q$^{\perp}$
max 38, sum 404
Tm1 → Dm3q → TmY9q$^{\perp}$
max 6, sum 33
Mi1 → Y3 → TmY9q$^{\perp}$
max 5, sum 38
Tm9 → T5a → TmY9q$^{\perp}$
max 5, sum 70
Mi9 → TmY11 → TmY9q$^{\perp}$
max 13, sum 85
Mi1 → T4a → TmY9q$^{\perp}$
max 8, sum 37
Tm1 → T2a → TmY9q$^{\perp}$
max 4, sum 39
Tm1 → TmY9q → TmY9q$^{\perp}$
max 7, sum 48
Tm1 → TmY9q$^{\perp}$ → TmY9q$^{\perp}$
max 5, sum 52
Mi4 → TmY10 → TmY9q$^{\perp}$
max 8, sum 52
Tm1 → Dm3v → TmY9q$^{\perp}$
max 0, sum 6
Tm1 → TmY9q$^{\perp}$ → Dm3q → TmY9q$^{\perp}$
